# Anti-Hu antibodies activate enteric and sensory neurons

**DOI:** 10.1038/srep38216

**Published:** 2016-12-01

**Authors:** Qin Li, Klaus Michel, Anita Annahazi, Ihsan E. Demir, Güralp O. Ceyhan, Florian Zeller, Lars Komorowski, Winfried Stöcker, Michael J. Beyak, David Grundy, Gianrico Farrugia, Roberto De Giorgio, Michael Schemann

**Affiliations:** 1 Human Biology, Technical University of Munich, Freising, Germany; 2 Department of Physiology, Shandong University School of Medicine, Jinan, China; 3 Department of Surgery, Klinikum Rechts der Isar, Technical University of Munich; Munich, Germany; 4 Surgery, Clinic Freising, Freising, Germany; 5 Institute for Experimental Immunology, Euroimmun AG, Lübeck, Germany; 6 GI Diseases Research Unit, Queen’s University, Kingston, ON, Canada; 7 Department of Biomedical Sciences, University of Sheffield, Sheffield, UK; 8 Enteric NeuroScience Program, Mayo Clinic, Rochester, MN, USA; 9 Department of Medical and Surgical Sciences and Center for Applied Biomedical Research, University of Bologna, Bologna, Italy

## Abstract

IgG of type 1 anti-neuronal nuclear antibody (ANNA-1, anti-Hu) specificity is a serological marker of paraneoplastic neurological autoimmunity (including enteric/autonomic) usually related to small-cell lung carcinoma. We show here that IgG isolated from such sera and also affinity-purified anti-HuD label enteric neurons and cause an immediate spike discharge in enteric and visceral sensory neurons. Both labelling and activation of enteric neurons was prevented by preincubation with the HuD antigen. Activation of enteric neurons was inhibited by the nicotinic receptor antagonists hexamethonium and dihydro-β-erythroidine and reduced by the P2X antagonist pyridoxal phosphate-6-azo (benzene-2,4-disulfonic acid (PPADS) but not by the 5-HT_3_ antagonist tropisetron or the N-type Ca-channel blocker ω-Conotoxin GVIA. Ca^++^ imaging experiments confirmed activation of enteric neurons but not enteric glia. These findings demonstrate a direct excitatory action of ANNA-1, in particular anti-HuD, on visceral sensory and enteric neurons, which involves nicotinic and P2X receptors. The results provide evidence for a novel link between nerve activation and symptom generation in patients with antibody-mediated gut dysfunction.

Neuro-immune interactions are thought to play a pathogenic role in patients with a variety of gastrointestinal neuromuscular or neuroepithelial disease^1^. In a subset of these patients, the inflammatory/immune insult is such as to define an enteric neuropathy, a term used to indicate the predominant involvement of the intrinsic innervation supplying the gut, i.e. the enteric nervous system (ENS)^2^. The classic histopathological correlate of inflammatory neuropathies is a dense infiltrate of CD3+ T lymphocytes (and, to a lower extent, plasma cells) localized within the two ganglionated plexuses of the ENS (hence the term ‘enteric ganglionitis’). For reasons that are still unclear, the inflammatory infiltrate more commonly affects myenteric (i.e. ‘myenteric ganglionitis’) rather than submucosal ganglia, although immune cell density is higher in the submucous and epithelial layers. Also, myenteric ganglionitis is usually accompanied by an inflammatory axonopathy, i.e. axons from myenteric neurons exhibit a lympho-plasmacellular infiltrate^3^. The inflammatory/immune-mediated changes within enteric ganglia and nerves can occur at any level of the gastrointestinal tract leading to severe gut dysmotility and delayed transit, detectable in conditions such as achalasia, gastroparesis, intestinal pseudo-obstruction and colonic inertia/megacolon. If unopposed by any pharmacological treatment (i.e., immunosuppressants), the inflammatory/autoimmune injury of the ENS can progress towards neuronal damage and loss with further deterioration of gut function.

In addition to the activation of immunocytes, patients with inflammatory neuropathies may develop a strong humoral response with a wide array of circulating anti-neuronal antibodies targeting molecules expressed by neurons, including the RNA binding protein Hu (anti-Hu also referred to as type-1 anti-neuronal nuclear antibodies or ANNA-1)^4^,^5^. Whereas HuA (HuR) is ubiquitously present, HuB, HuC and HuD are specifically expressed in neurons and located in the nucleus or cytoplasm. However, it is the HuD antigen that is most frequently produced by small cell lung cancer cells. In most cases anti-HuD antibody associated syndromes occur with lung cancer, in particular small cell lung cancer. Although anti-neuronal antibodies can be sometimes found in the sera of patients with idiopathic ganglionitis, they are usually detected in cases of gut motor disorders associated with paraneoplastic syndromes^6^,^7^. The detection of anti-neuronal antibodies can be useful to guide an appropriate diagnostic approach, but their pathogenic role in ENS damage is still unsettled. It can be tentatively speculated that the activation of the immune system facilitates the access of immunocytes to the ENS microenvironment, as reflected by the histopathological changes found in enteric ganglionitis^3^. This would allow direct exposure of enteric neurons to IgGs. Incubation of cultured myenteric neurons with Hu-positive sera from patients with paraneoplastic gut dysmotility as been shown to cause apoptosis^8^. Similar damage to enteric neurons may occur in patients with irritable bowel syndrome (IBS), a condition characterized by a cluster of symptoms such as abdominal pain and bowel habit changes, who have circulating antibodies against enteric neurons^9^. The pathological effects of autoantibodies vary according to the target antigen, but neuronal dysfunction may be reversed with antibody-depleting therapies^10^. Although there is consensus about the neuronal damage or loss of function evoked by chronic exposure to some circulating antibodies, the acute effects of autoantibodies on neuronal function is unknown.

Thus the aim of the present study was to determine the effect of sera from paraneoplastic syndrome patients with elevated ANNA-1 level on action potential discharge of human and guinea-pig enteric neurons as well as on mice visceral afferent nerves. We also tested if the purified IgG fractions or the purified HuD-antibody were able to mimic the effect of the sera.

## Materials and Methods

For the study we used human serum samples and human and guinea pig tissue samples. The experimental protocols were approved by the The Mayo Clinic institutional review board (IRB # 08-005481 for sampling of sera) and the Ethic Committee of the Technical University of Munich (Project approval 1746/07 and 1512/06 for tissue sampling). Patients gave written informed consent. All studies were conducted in accordance with the Declaration of Helsinki (Prot.n. 906/2006 and Prot.n 1925/2004) and methods were carried out in accordance with approved guidelines.

All animal studies were carried out in accordance with the German guidelines for animal protection and animal welfare and with approval of the local animal ethical committee at district veterinary office of Munich.

### Preparation of purified Immunoglobulin G fractions (IgGs)

IgGs were purified from sera of seven patients with paraneoplastic syndrome evoking gut dysfunction (i.e., a severe panenteric motility disorder such as chronic intestinal pseudo-obstruction) and high ANNA-1 titers (Table 1) and a group of 8 age- and sex-matched healthy controls. The ANNA-1 containing patient sera tested negative for antibodies against voltage-gated potassium channels, N-type calcium channels, P/Q-type calcium channels, muscle nicotinic acetylcholine receptors (α1 subunit), neuronal nicotinic acetycholine receptors (α3 subunit), collapsin response-mediator protein 5, glutamic acid decarboxylase, Purkinje cell cytoplasmic autoantibody type 1 and striational antibodies^11^.

### Isolation of anti-HuD autoantibodies (anti-HuD)

#### Preparation of recombinant HuD

Human HuD (Acc. NM_021952) was expressed in E. coli BL21 as previously described^12^. Full length cDNA coding for HuD was ligated into pET24d (Novagen) in frame with the coding sequence for a C-terminal histidyl octamer to allow metal chelate chromatography purification. Expression was obtained using the pET system standard conditions. Recombinant HuD-His was purified from inclusion bodies by differential centrifugation, extraction in 5 mM tris-HCl pH 8, 300 mM NaCl, 8 M urea, and subsequently metal chelate chromatography on NiNTA sepharose (Qiagen). The protein was eluted in 50 mM sodium acetate pH 4.5, 8 M urea.

#### Coupling of recombinant HuD to NHS activated sepharose

Purified, denatured HuD-His was mixed with four volumes of refolding buffer (200 mM ethanolamine pH 10, 500 mM NaCl with 50% (w/v) glycerol) and dialyzed against coupling buffer (200 mM NaHCO_3_, pH 8.3, 500 mM NaCl). Insoluble material was removed by filtration with a 0.2 μm filter. A 1 ml HiTrap NHS column (GE Healthcare, Freiburg, Germany) was washed with ice-cold HCl (1 mM) and coupling buffer and then loaded with 1.5 mg soluble HuD-His in 1 ml coupling buffer for 30 min at room temperature. After 3 washes with high pH buffer (500 mM ethanolamine pH 8.3, 500 mM NaCl) and low pH buffer (500 mM sodium acetate pH 4, 500 mM NaCl), respectively, the column was finally washed with PBS.

The HuD-column was used to purify anti-HuD autoantibodies from the serum of a patient with chronic intestinal pseudo-obstruction of non-paraneoplastic origin (Table 1, #27)^8^. This serum was used to obtain purified human anti-HuD. The serum (anti-Hu 1:1000) was 1:4 diluted with PBS, sterile filtered and passed onto the HuD-column. After washing the column with PBS (10 ml) anti-HuD was eluted with 200 mM glycine at pH 2.5. Eluate fraction was neutralized with one volume of 7.5% (w/v) Na_2_CO_3_) and then dialyzed five times against 10 volumes PBS. The titer of the purified anti-HuD was 1:320.

### Immunohistochemistry

Tissue specimens of adult male guinea pigs (weight 250 g–550 g) ileal longitudinal muscle myenteric plexus preparations were fixed overnight at room temperature in a solution containing 4% paraformaldehyde and 0.2% picric acid in 0.1 M phosphate buffer and then washed (3 × 10 min) in phosphate buffer. After 1 hr incubation in PBS plus Triton-X-100 (TX100; 0.5%) and horse serum (4%), the patient #27 serum, purified IgG control and patient samples (1:500 each), purified human anti-HuD (from patient #27, 1:5,000–1:20,000), mouse anti-HuC/D (1:50, Thermo Fisher Scientific, Waltham, MA, USA), goat anti-CD64 (label for Fcγ receptor, 1:200, Santa Cruz Biotechnology, Heidelberg, Germany), or rabbit anti-neuron-specific enolase (NSE, 1:3000, Polysciences, Eppelheim, Germany) were added for 12–16 hrs. After washing (3 × 10 min in PBS) the preparations were incubated for 6 hours with species specific secondary antibodies coupled to fluorophores (all from Dianova, Hamburg, Germany); Cy3-conjugated anti human or anti goat IgG (1:500, Dianova, Hamburg, Germany), Dylight 488 conjugated anti-rabbit or anti-human IgG (1:200), or streptavidin-Cy3 conjugated anti-mouse IgG (1:200). After the final washing, the tissues were mounted on poly-l-lysine coated coverslips and evaluated with an Olympus Microscope BX61 WI (Olympus, Hamburg, Germany) with appropriate filter blocks. The microscope was equipped with a SIS Fview II CCD-camera and the analySIS 3.1 software (Olympus Soft Imaging Solutions GmbH, Münster, Germany) for image acquisition and analysis. The specificity of the purified anti-HuD was tested by preincubation of the antibody with the HuD antigen^13^ or a fragment of the antigen containing the amino acids (aa) 90–101 (gfvnyidpkdae) (Department of Molecular Biology and Biotechnology, University of Sheffield, UK). This fragment represents the epitope recognized by all anti-HuD containing patient sera^14^. For these experiments the antibody was used at a dilution of 1:20,000 which corresponded to ~26 pmol/l anti-HuD IgG. The HuD antigen was added at concentrations between 2.4 and 480 nmol/l and the 90–101aa HuD fragment between 0.44 and 440 μmol/l. After overnight incubation of antibody and antigens, the mixture was used for staining as described above.

### Tissue preparation and voltage or Ca^++^ sensitive dye imaging

The imaging techniques to record neural activity in the ENS were previously described^15^,^16^,^17^. Human tissue was obtained from patients undergoing abdominal surgery in the Departments of Surgery at Clinic Freising and Clinic Rechts der Isar. Samples were taken from macroscopically normal, unaffected areas as determined by visual inspection of the pathologist. Tissues were placed in cold oxygenated sterile Krebs solution containing (in mM): 117 NaCl, 4.7 KCl, 1.2 MgCl2 6H_2_O, 1.2 NaH_2_PO_4_, 25 NaHCO_3_, 2.5 CaCl_2_ 2H_2_O and 11 glucose (all chemicals from Sigma-Aldrich). After arriving in the laboratory intestinal tissues were further dissected in ice-cold sterile oxygenated Krebs solution to obtain a preparation of the intact inner submucous plexus. Ileum preparations from adult male guinea pigs (weight 250–550 g) were obtained after killing the animals by cervical dislocation. The tissues were further dissected to obtain longitudinal muscle/myenteric plexus or lamina propria/submucous plexus preparations.

The final preparations were placed in the recording chamber and continuously perfused with 37 °C Krebs solution. The tissue chamber with the guinea pig or human preparation was mounted onto an epifluorescence Olympus IX 50 microscope (Olympus, Hamburg, Germany) equipped with a 150 W xenon arc lamp (Osram, Munich, Germany). In the majority of experiments illumination periods of 1.3–5 sec (potentiometric dye) or up to 30 sec (Ca^++^ sensitive dye) were used, which were sufficient to record representative responses. Enteric neurons were stained with the fluorescent voltage sensitive dye Di-8-ANEPPS (1-(3-sulfonatopropyl)-4-[β[2-(di-n-octylamino)-6-naphthyl]vinyl]pyridinium betaine, life technologies, Darmstadt, Germany) or Fluo4-AM. Di-8-ANEPPS or Fluo4-AM stained preparations were visualized with oil immersion objectives (UAPO/340 Olympus, Hamburg, Germany) and neural activity was recorded at a single cell level with a photodiode or CCD based system, respectively (NeuroPDA or NeuroCCD system, RedShirt Imaging, Decatur, GA, USA). We tested responsiveness of neurons by evoking fast excitatory postsynaptic potentials (fast EPSPs) after electrical stimulation of interganglionic fiber tracts with a monopolar platinum electrode or by pressure ejection of nicotine. Raw signals were converted to relative changes in fluorescence (∆F/F) and analyzed with Neuroplex (RedShirt Imaging) or Fiji^18^.

Sera or purified IgGs were applied to single ganglia by pressure ejection from micropipettes (20 psi, 400 ms duration, 200 μm distance to the ganglion). Any substance in the microejection pipette was diluted by a factor of approximately 10 before reaching the ganglion^19^. Antagonists were added directly to the superfusing Krebs solution. We used the non-selective nicotinic receptor antagonist hexamethonium (100–200 μM, Sigma-Aldrich), the β2 subunit preferring nicotinic receptor antagonist dihydro-β-erythroidine (10 μM, Sigma-Aldrich), the 5-HT_3_ receptor antagonist tropisetron (0.1 μM, gift from Sandoz), the P2X receptor antagonist pyridoxal phosphate-6-azo (benzene-2,4-disulfonic acid) tetrasodium salt hydrate (PPADS, 10 μM, Sigma-Aldrich) and the N-type Ca^++^ channel blocker ω-Conotoxin GVIA (60 nM, Alomone labs, Israel).

### Afferent recordings

These experiments were performed at the University of Sheffield under licence from the UK Animals Scientific Procedures Act (1986). Experiments were performed in tissues from 14 C57Bl6 mice of either sex (8–12 weeks old, 20–30 g). Mice were killed by cervical dislocation and the stomach and distal esophagus were removed into oxygenated (95% O_2_ and 5% CO_2_; pH = 7.40) Krebs solution. The gastric fundus together with the distal esophagus were isolated, thoroughly cleaned with Krebs solution and opened along the lesser curvature to obtain a flat sheet preparation. The mucosa was removed and fine branches of the vagus nerve innervating the fundus were preserved. Vagal multifibre afferent discharge was recorded with extracellular suction electrode (tip diameter 50–100 μm) as previously described in detail^20^,^21^. The electrode was connected to a Neurolog headstage (NL100, Digitimer, Welwyn Garden City, UK) and the signal was amplified (NL104, Digitimer) and filtered with a band pass 200–3000 Hz (NL 125, Digitimer). The nerve signal was acquired (20 kHz sampling rate) through a Micro 1401 interface (CED, Cambridge Electronic Design, Cambridge, UK) and analyzed using Spike2 software (version 5.03, CED). In addition to the raw nerve signals, afferent fibers activity was recorded as a rate histogram with spike discriminating software to identify single units. Mechanosensitive receptive fields were located by probing the serosal surface with von Frey filaments (tip diameters <50 μm), exerted a force of 1–2 mN. In some preparations the buffer was switched to a calcium-free Krebs with MgCl_2_ at a concentration of 3.6 mmol/L. Chemical stimulation was achieved via a silicone ring (approximately 100 μl volume) placed over the receptive ‘hot spot’. Afferent units first were initially tested for their sensitivity to the nicotinic receptor agonist 1, 1-dimethyl-4-phenylpiperazinium iodide (DMPP, 100 μmol/l, Sigma) and/or adenosine 5′-triphosphate (ATP, 100 μmol/l, Sigma). Then anti-HuD sample (patient #27) was applied. The anti-HuD sample was stored at 4 °C in aliquots and diluted 1:10 with Krebs solution just before the experiments.

### Data analysis and Statistics

Individual enteric neurons can be visualized in the neuroimaging experiments because the voltage sensitive dye incorporates into the outer neuronal membrane, thereby revealing the outline of individual cell bodies. This allowed us to determine the total number of neurons in each ganglion. For analysis, the optical signals were superimposed onto the image of the ganglion. For each ganglion, the total number of neurons and the number of neurons showing a response to the application of the compounds were calculated. Data were expressed as number of responding neurons vs. number of all neurons and mean frequency ± standard error or median frequency [Q_0.25_/Q_0.75_] over all responding neurons. The neuroindex was calculated by multiplying the percentage of responding neurons with the mean or median response frequency. Differences between these data were tested with t-tests/rank sum tests or one way ANOVA/repeated measures ANOVA on rank. Differences in the number of responding neurons were tested with χ^2^-test/Fisher exact test or McNemar test (for repeated measures). Correlations were tested with the Pearson Product Moment correlation test. A *P* value < 0.05 was considered statistically significant. For multiple comparisons we applied a Bonferroni correction. The software for statistical calculations was SigmaPlot 12.5 (Systat Software Inc., Erkrath, Germany) or Igor Pro 6.3 (Wavemetrics, Lake Oswego, OR, USA). Afferent neurograms were analyzed using Spike2 software to calculate the firing rate of spikes crossing a preset threshold in sequential time bins. The gastric vagal afferent activity was expressed as mean spikes frequency over the period of the response. Peak discharge rate was determined over a 3 s period around the maximal response. The latency was measured from sample application to a 20% increase in firing rate above baseline activity or when a peak was reached. Statistical analysis of neurogram data was performed using GraphPad Prism version 4.01 (GraphPad Software, San Diego, CA, USA). Data are expressed as medians and compared using Wilcoxon Signed Rank Test.

## Results

### Purified patient IgGs (ANNA-1 IgGs) and purified anti-HuD labeled enteric neurons

**Figure 1:**
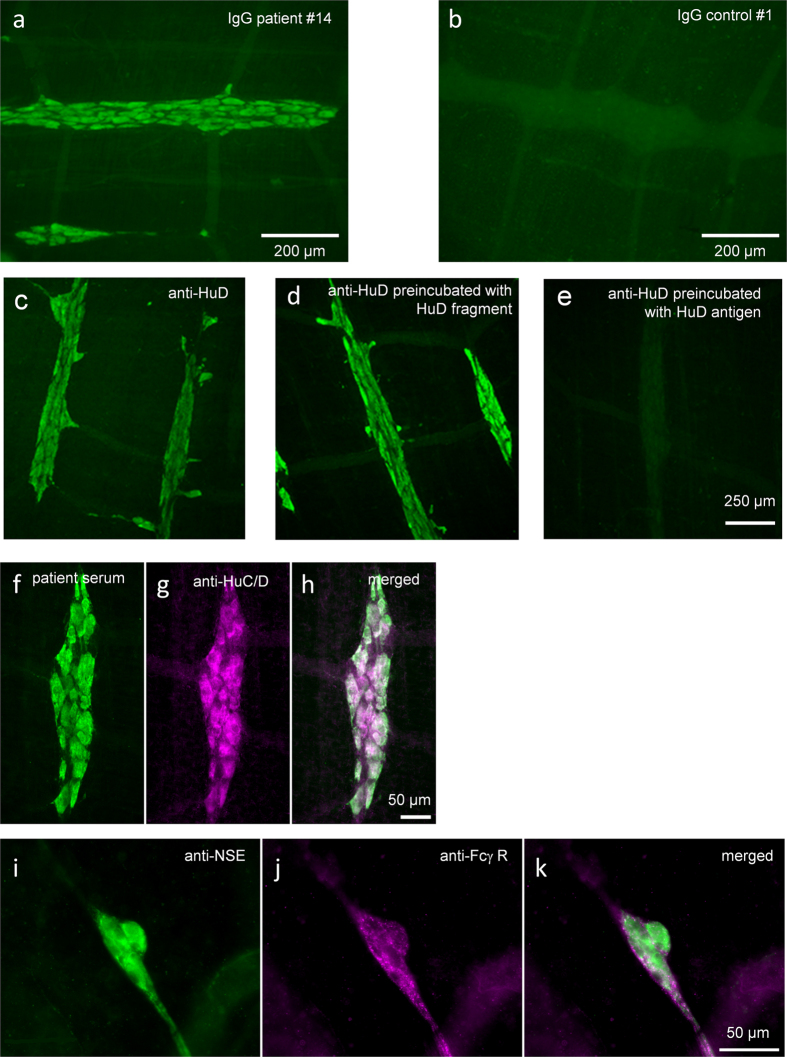
ANNA-1 sera and purified IgG fractions label guinea pig enteric neurons. Purified IgGs from patients with high ANNA-1 titer (**a**), but not IgGs from healthy controls (**b**), strongly label nerve cell bodies in the guinea pig myenteric plexus. Purified anti-HuD also reveals staining of enteric neurons (**c**), which remains after preincubation with the HuD fragment aa90-101 (**d**) but is blocked by preincubation with the HuD antigen (**e**). Labeling of enteric nerve cell bodies by whole serum is similar to staining with patient IgGs or purified anti-HuD (**f**). This labeling is identical to that obtained with a commercial HuC/D antibody (**g**) and merged image in (**h**). Anti-NSE labels nerve cell bodies and fibers in a human submucous ganglion (**i**). FCγ receptor staining with CD64 antibody labels nerve fibers only in the same ganglion (**j**) and merged image in (**k**). Serum was diluted 1:10,000, purified IgGs from patient or control 1:500 and purified anti-HuD 1:20,000; see methods for other antibodies.

We assessed the ability of patient #27 serum, purified IgGs from patient or control sera and purified anti-HuD to bind to targets in the myenteric plexus of the guinea-pig ileum. All samples, except those from healthy controls, revealed strong staining of enteric cell bodies but no staining of neuronal processes (Fig. 1). While 6 samples stained nuclei and somata, one sample (#2) showed labeling of neuronal nuclei only. The eight purified IgG samples from healthy controls showed no staining of enteric neurons or any other structure in the preparations. Importantly, staining with the purified anti-HuD was identical to the staining pattern observed with the whole serum or the purified patient IgG samples. Preincubation with the HuD antigen in excess of 1,800 or 18,000 reduced or blocked the staining by the purified anti-HuD. However, staining by anti-HuD was not affected by preincubation with the aa90–101 HuD fragment even at concentrations that correspond to a 17 million fold excess of the fragment over the antibody.

### Purified patient IgGs (ANNA-1 IgGs) activated enteric neurons

**Figure 2:**
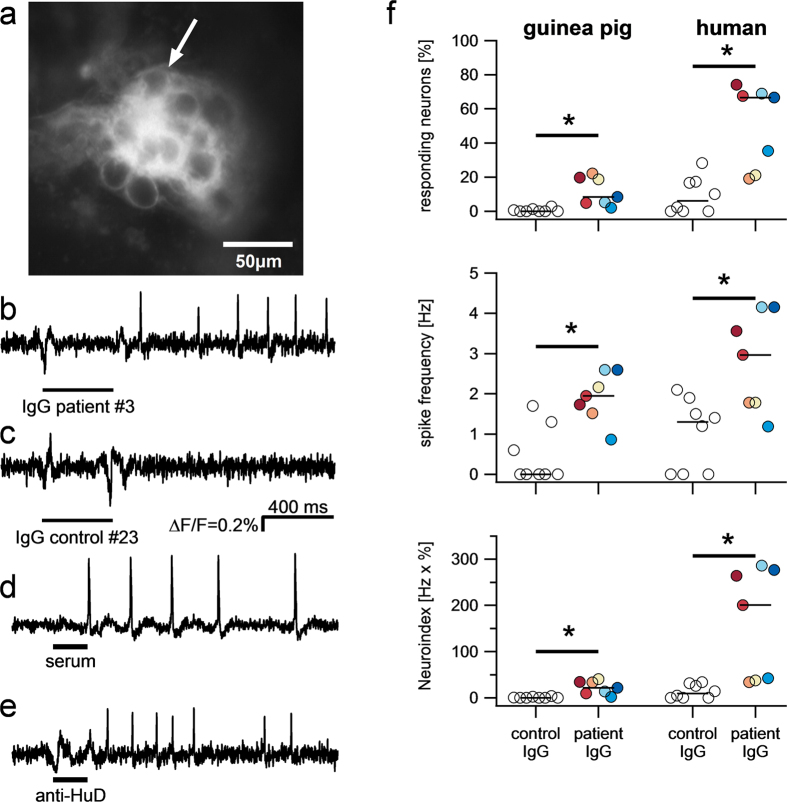
Purified IgG from patients but not from healthy controls activate enteric neurons. High resolution image of a ganglion in the human submucous plexus after incubation with the voltage sensitive dye Di-8-ANEPPS (**a**). The dye incorporates into the outer membrane and reveals the outline of nerve cell bodies. Example traces of one of the neurons (marked by arrow) show a fast onset spike discharge after brief spritz application (bars below traces) of purified IgG from patient #3 (**c**) but no response to IgG from healthy control # 23 (**d**). The serum of patient #27 (**e**) and the purified anti-HuD from this patient (**f**) both also evoke an immediate spike discharge. The degree of nerve activation is shown in panels g-I. They reveal that the patient IgGs evoked a significantly stronger spike discharge in a larger proportion of guinea pig (GP) and human enteric neurons. As a result the neuroindex was also significantly higher. Open circles are results based on application of purified IgG from 8 healthy controls in 46 ganglia and 1296 neurons from 18 guinea pig myenteric plexus or 33 ganglia and 258 neurons from 23 human submucous plexus preparations. Color-filled circles represent results from application of purified IgG from 7 patients in 57 ganglia and 1526 neurons from 23 guinea pig myenteric plexus or 36 ganglia and 304 neurons from 22 human submucous plexus preparations.

We first studied the effects of purified IgGs on myenteric neurons of the guinea pig ileum. Pressure application of the patient IgGs via a pipette located directly above the ganglia resulted in excitation of a subpopulation of neurons (Fig. 2). Action potential discharge occurred after a short latency and usually lasted throughout the recording period of several seconds. The excitatory response consisted of a fast onset action potential discharge or an increase in spike discharge in spontaneously spiking neurons. The excitatory effect of the ANNA-1 samples was significantly stronger than the effect of the control samples (Fig. 2). When we analyzed the responses to two repeated applications of the same ANNA-1 sample (performed for samples from patients #9, #10, #20) we found no significant differences in the number of responding neurons (25 out of 62 vs. 22 out of 62, p = 0.6, 3 tissues/3 ganglia/62 neurons) or spike frequency (1.4 ± 1.3 Hz vs. 1.2 ± 1.5 Hz, p = 0.46) between the 2 applications.

The nerve activation was evident at 1:100 dilutions but more robust effects were seen with 1:1 dilutions. The patient ANNA-1 titer to achieve staining was much higher than 1:1 and the IgG concentration in control samples was as high as in patient serum (Table 1). It was therefore not surprising that there was poor correlation between total IgG content or ANNA-1 titer and the percentage of responding neurons (total IgG: r = −0.52, P = 0.23, titer: r = −0.32, P = 0.49), spike frequency (total IgG: r = 0.24, P = 0.60, titer: r = 0.69, P = 0.08) or neuroindex (total IgG: r = −0.40, P = 0.37, titer: r = −0.14, P = 0.76).

Since recording of nerve activity in the human myenteric plexus is not technically feasible, we instead examined the effect of the purified IgGs from patients or controls on human submucous neurons (72 specimens from 41 donors). Here, we also found excitatory effects which were much stronger for patient than for control IgGs (Fig. 2). Repeated applications of patient IgGs resulted in comparable responses (61 out of 101 vs. 58 out of 101 responding neurons, P = 0.6; spike frequency of 2.4 ± 1.9 Hz vs. 2.8 ± 2.5 Hz, P = 0.09, 7 tissues/10 ganglia/101 neurons). Similar to our findings in guinea pig tissue, we found no correlations between total IgG content or antibody concentration (titer) and the percentage of responding neurons (total IgG: r = 0.38, P = 0.40, titer: r = 0.30, P = 0.51), action potential frequency (total IgG: r = 0.33, p = 0.47, titer: r = 0.47, P = 0.29) or neuroindex (total IgG: r = 0.35, P = 0.44, titer: r = 0.40, P = 0.37).

The nerve activating effect of the patients IgGs was more pronounced in human submucous neurons than guinea pig myenteric neurons. Both the number of responding neurons (11.6 ± 8.3% vs. 50.4 ± 24.3%, P = 0.01) and the spike frequency (1.8 ± 0.6 Hz vs. 2.8 ± 1.2 Hz P = 0.03) was higher in human submucous neurons. Samples which evoked a higher spike frequency in guinea pig enteric neurons, also had a stronger effect in human enteric neurons (r = 0.8, P = 0.036).

The complete serum (#27) activated guinea pig myenteric neurons in a comparable manner to that observed after application of the patient IgGs (Fig. 2). The nerve activation occurred in 224 out of 643 neurons (3 tissues/17 ganglia) and evoked a median spike discharge of 3.1 Hz [1.6/4.7]. Activation of human submucous neurons by the complete serum (#27) occurred in 35 out of 123 neurons (12 tissues/14 ganglia) with a median spike frequency of 2.4 Hz [1.2/4.0].

**Figure 3:**
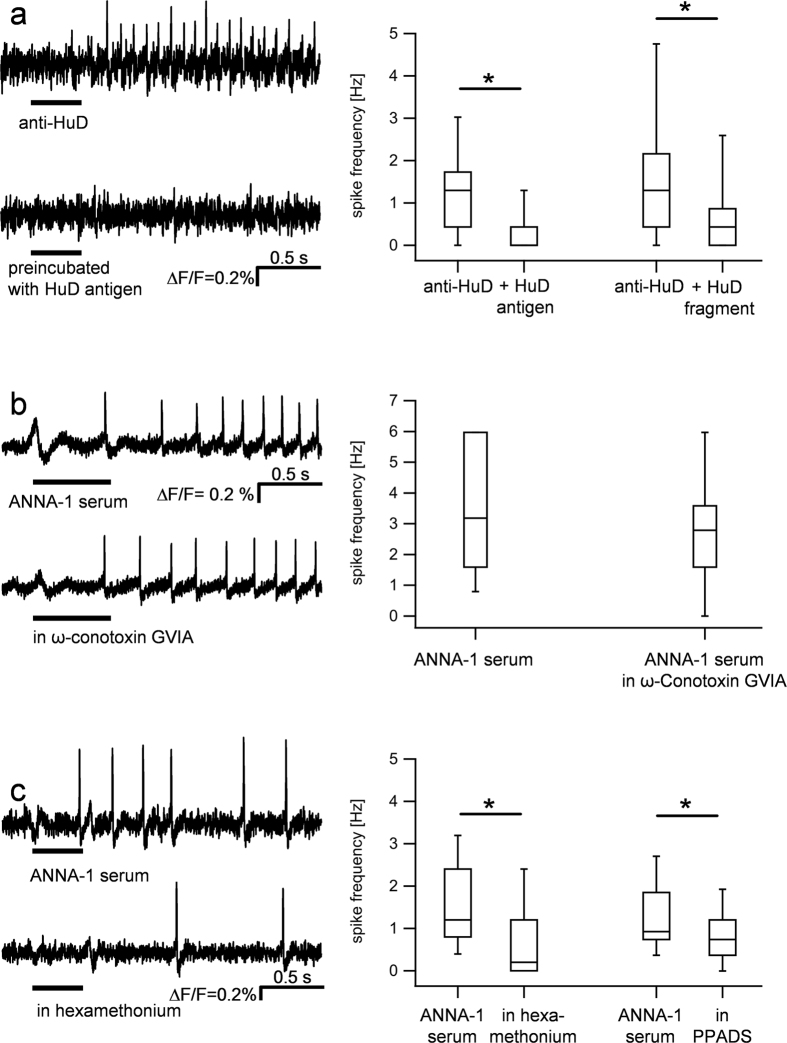
Purified IgGs evoked nerve activation is caused by direct postsynaptic activation of neurons and prevented or inhibited by preincubation with HuD antigen, the nicotinic receptor blocker hexamethonium and the P2X purine receptor antagonist PPADS, respectively. Application of anti-HuD evoked activation of guinea-pig enteric neurons (upper trace (**a**). The spike discharge was prevented by preincubation with the full HuD antigen (lower trace (**a**) and with the HuD fragment aa90-101. The graph summarizes the results after preincubation with HuD antigen (166 neurons, 19 ganglia from 9 guinea pig myenteric plexus preparations) and HuD fragment aa90-101 (39 neurons, 4 ganglia from 3 guinea pig myenteric plexus preparations). The response to serum from patient #27 (upper trace (**b**) is not affected by the N-type Ca^++^ channel blocker ω-conotoxin GVIA (lower trace (**b**) which also blocks all synaptic input (statistics based on 7 neurons, 3 ganglia and 3 human submucous plexus preparations). However, the response to serum from patient #27 (upper trace (**c**) is strongly reduced by hexamethonium (lower trace (**c**) and by the P2X purine receptor antagonist PPADS (statistics based on 111 neurons, 10 ganglia, 8 guinea pig myenteric plexus preparations for hexamethonium and 18 neurons, 4 ganglia, 2 guinea pig myenteric plexus preparations for PPADS).

The purified anti-HuD mimicked the effects of the serum of this patient (#27) and the effects evoked by the patient IgGs. Anti-HuD evoked a spike discharge of 2.2 Hz [1.3/3.5] in 38% of guinea pig myenteric neurons (226 out of 863 neurons) (Fig. 3).

In order to determine if the anti-HuD induced nerve activation was mediated by the binding of the paratope to a neuronal target, we tested whether preincubation with the HuD antigen or 90–101aa HuD fragment prevented the effect. The anti-HuD IgG (dilution 1:1, ~1 μmol/l) activated 134 out of 484 neurons which fired at 1.3 Hz [0.4/1.7]. Preincubation with the HuD antigen (67 μM/l) significantly reduced the number of responding neurons (37 out of 484, P < 0.0001) and the spike frequency (0.0 Hz, [0.0/0.4], P < 0.001). Preincubation of anti-HuD (dilution 1:10, ~0.1 μM) with the 90–101aa HuD fragment (6.7 μM) similarly reduced the nerve activation by anti-HuD. While 35 out of 131 neurons fired after anti-HuD at a frequency of 1.3 Hz [0.4/2.2] the number of responding neurons as well as the spike frequency decreased after preincubation with the HuD fragment to 22 out of 131 (p = 0.009) and 0.4 Hz [0.0/0.9] (P < 0.001), respectively.

**Figure 4:**
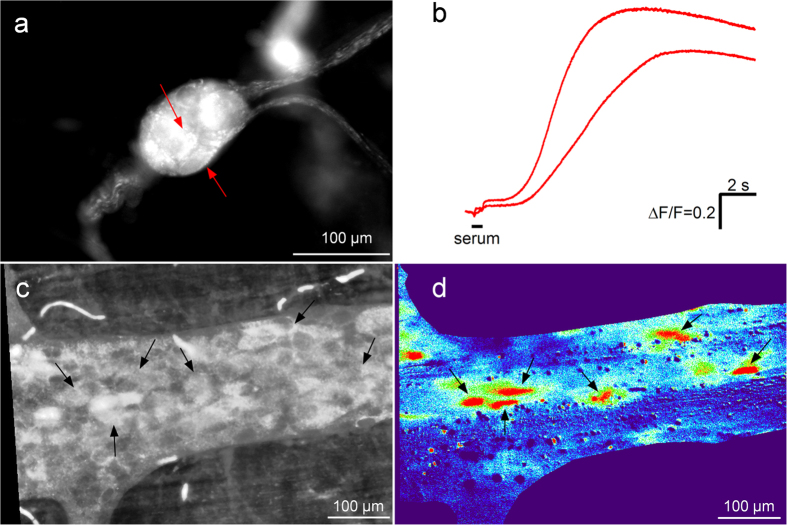
ANNA-1 serum from patient #27 evokes neuronal Ca^++^ responses. ANNA-1 serum specifically activates neurons in the human submucous (**a** and **b**) and the guinea-pig myenteric plexus (c and d). Panel a shows a human submucous ganglion after NSE staining which labels only neurons. The Ca^++^ traces of two neurons (marked by red arrows) following application of serum #27 are shown in panel B (application of serum indicated by the bar). Panel c shows a guinea pig myenteric ganglion after NSE staining. The Ca^++^ responses of the neurons marked by black arrows are shown in panel d as color coded signals 13 sec after application of the serum (red = high activity – blue = no activity).

Because the excitatory effect of anti-HuD could be mediated by pre- or postsynaptic targets, we applied anti-HuD IgG in the absence and presence of ω-Conotoxin GVIA (60 nM) in preparations of the human submucous plexus. ω-Conotoxin GVIA is a blocker of N-type calcium channels that blocks electrically induced synaptic responses in the ENS when recorded with voltage sensitive dye imaging^17^,^22^, Ca^++^ imaging^23^ or intracellular electrodes (virtually abolished)^24^. We found no significant difference in the response to anti-HuD before and after ω-Conotoxin GVIA (spike frequency 3.4 ± 2.9 Hz vs. 2.6 ± 1.9 Hz, P = 0.162), suggesting a direct, postsynaptic effect of the antibodies (Fig. 3). These findings argued against presynaptic action of the sera. However, we also wanted to rule out the possibility that neural spiking was a consequence of glia activation. We therefore performed Ca^++^ imaging in guinea-pig myenteric and human submucous neurons followed by immunohistochemical staining for the neuronal marker NSE (Fig. 4). Application of serum #27 to guinea pig myenteric neurons resulted in a Ca^++^ signal in 41% of the cells (33 out of 81) with an increase in Ca^++^ of 25.2% [21.2/38.7] ∆F/F. Similar results were obtained in human submucous plexus where 50% of the cells (14 out of 28) responded with an increase in Ca^++^ of 28% [24.0/46.5] ∆F/F. Those ganglion cells which responded to the ANNA-1 serum were neurons because they were all NSE immunoreactive or responded to nicotine which evoked immediate activation only in enteric neurons but not glia (Fig. 4).

The fast onset response after application of the serum #27, the patient IgGs as well as the purified anti-HuD was striking. We therefore analysed in detail the delay between the beginning of the pressure application and the first action potential. In guinea pig preparations the first action potential in response to application of purified anti HuD, serum #27 and ANNA-1 sample #3 occurred after 316.9 ms [198.2/528.4] (n = 38 neurons), 425.4 [300.4/731.3] (n = 27 neurons) and 453.0 ms [357.7/682.9] (n = 13 neurons). The latency after nicotine application was not different and the first action potential occurred at 349.4 ms [185.1/883.9] (n = 27 neurons, P = 0.08). This lack of significant difference in latency was true also in human preparations (P = 0.44). The first spike after application of nicotine, purified anti HuD or ANNA-1 sample #3 occurred after 251.3 ms [194.8/577.1] (n = 20 neurons), 442.9 ms [287.8/520.7] (n = 14 neurons) or 310.2 ms [213.9/720.3] (n = 27 neurons), respectively. These data suggest that ionotropic receptors may be one of the targets of anti-HuD; in the ENS the most prominent among them are nicotinic, P2X and 5-HT_3_ receptors^25^,^26^. We found that hexamethonium significantly reduced the response to the serum #27 as indicated by the reduced number of responding neurons (37 out of 102 neurons vs. 9 out of 102 in hexamethonium, P < 0.001) and the decreased spike frequency (1.2 Hz [0.8/2.4] vs. 0.2 Hz [0.0/1.2] in hexamethonium, P < 0.001) (Fig. 3). Similarly, hexamethonium decreased the spike frequency evoked by the IgG samples of patients #2, 8 and #10 (only those were used) from 1.2 Hz [0.4/2.1] to 0 Hz [0.0/0.0], P = <0.001, n = 14 neurons]. The nerve activation by purified anti-HuD was also reduced by hexamethonium and in addition by DHβE from 1.3 Hz [0.8/2.0] to 0.4 Hz [0.0/1.88] (n = 19 neurons, P = 0.002) and 1.2 Hz [0.4/3.0] to 1.0 Hz [0.3/1.6] (P = 0.048, n = 22 neurons), respectively. The P2X antagonist PPADS (10 μM) reduced the action potential frequency in response to serum #27 significantly from 1.3 ± 0.9 Hz to 0.8 ± 0.7 Hz (n = 18 neurons, p = 0.004). In contrast, we did not find evidence for involvement of 5-HT_3_ receptors. The 5-HT_3_ antagonist tropisetron (0.1 μM) did not change the IgG evoked nerve activation (spike frequency: 1.2 ± 0.8 Hz vs. 1.4 ± 1.2 Hz, p = 0.3, n = 6 neurons).

### Afferent recordings

**Figure 5:**
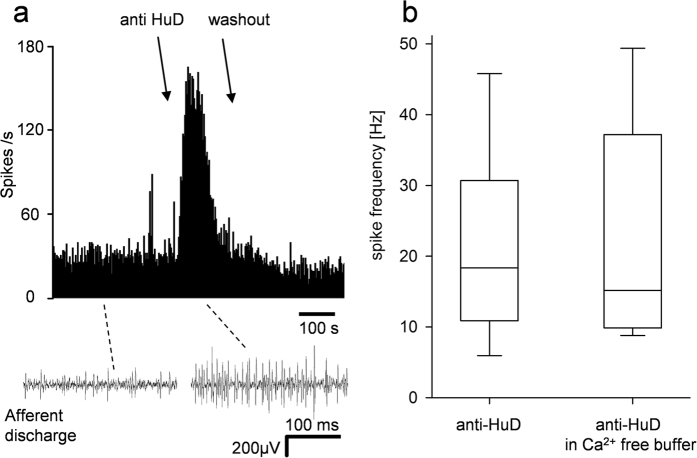
Purified anti-HuD augments spike discharge in visceral sensory nerve fibers. Panel **a** is an example of a sequential rate histogram showing an increase in spike discharge shortly after application of the anti-HuD. The traces below are sample neurograms showing the spike discharge before and after anti-HuD. The graph in **b** summarizes results from several such experiments and revealed a significant increase in spike discharge rate by anti-HuD (recordings from 20 afferent nerves in 15 mouse stomach preparations) which was not affected in Ca^++^ free buffer (recordings from 9 afferent nerves in 6 mouse stomach preparations).

Purified anti-HuD (dilution 1:10) was applied to 15 mechanosensitive “hot spots” from 10 mice and a total of 20 units were analyzed. 18 out of 20 units (90%) responded with a significant increase in firing rate by 18.3 Hz [11.0/27.5] (mean latency 25.9 ± 4.5 s, peak after 75.8 ± 10.7 s) (Fig. 5). The response rapidly desensitized (t1/2 = 10.2 s) but in some cases was sustained until washing out the sample. This effect was reproducible based on the finding that repeated application of anti-HuD to 4 mechanosensitive fibers evoked similar spike frequencies (first application 9.9 Hz vs 11.3 Hz after second application of anti-HuD, p = 0.82). Vagal afferents responded to DMPP and/or ATP but sensitivity to anti-HuD was not correlated to either. A substantial number of vagal afferents terminate in enteric ganglia raising the possibility of activation secondary to an action on enteric neurons. However, the response to anti-HuD was not significantly different in calcium-free buffer from that in standard Krebs solution (18.3 Hz [11.0/27.5] n = 18 vs. 15.2 Hz [9.9/33.6] n = 9, p = 0.78). This finding suggested that the anti-HuD evoked increase in spike discharge was indeed mediated by direct activation of afferent fibers rather than by indirect activation followed by the release of excitatory enteric neurotransmitters. This was the case for both phasic and sustained components of the afferent response (Fig. 5).

## Discussion

We demonstrated that ANNA-1 autoantibodies from patients with paraneoplastic GI syndromes labelled all neurons in the myenteric plexus of the guinea pig. In functional studies, ANNA-1 autoantibodies had an excitatory effect on human and guinea pig enteric neurons as well as on mouse visceral afferents, which was reproducible upon repeated applications. Using receptor antagonists we reveal that both nicotinic (β2 subunit) and purinergic P2X receptors are implicated in the responses to ANNA-1 autoantibodies. Activation of enteric or visceral afferent nerves was likely to be a direct effect because the spike discharge did not change after synaptic blockade by Ca-depletion or ω-conotoxin GVIA. We found that anti-HuD was responsible for this excitatory effect because it mimicked the excitation evoked by purified IgGs, which was reversed by the HuD antigen. This is the first demonstration that ANNA-1 antibodies caused fast onset activation of enteric neurons. These acute effects of ANNA-1 antibodies contrast with several long term effects of anti-neuronal antibodies which all inhibit excitability, impair neuronal functions or even irreversibly damage or kill neurons^27^,^28^,^29^,^30^.

Although the most-established role of HuD is in neurogenesis, it also plays a role in neuronal survival, plasticity during learning and memory, and following neuronal injury. Despite HuA, HuB, HuC and HuD proteins sharing more than 80% homology, there are some noteworthy differences, which suggest unique functions of HuD. Thus, the spatial expression of HuD mRNA and the amino acid sequence in the N-terminal region of HuD are different from the other Hu proteins^31^. Although loss of function of Hu proteins impact on neuronal signaling because of impaired RNA stabilization, there is so far no evidence for direct involvement of HuD in neuronal excitability.

Only a few studies have shown direct activation of neurons in response to application of antibodies^32^,^33^,^34^,^35^. An antibody directed against the glutamate receptor 3B protein activated hippocampal neurons^28^. In two other studies, binding of IgG immune complexes to neuronal Fcγ receptors activated ion channels leading to depolarization^32^,^34^. Finally, neuronal activation occurred after application of antibodies against dipeptidyl-peptidase-like protein 6, a subunit of Kv4.2 potassium channels^33^. Other antibodies that excited enteric neurons were anti-idiotypic antibodies to 5-HT receptors^35^. In all studies the targets of the particular antibodies were a well-defined accessible receptor or ion channel linked to excitability of neurons. It is not clear how the anti-HuD antibodies activated neurons because the only known epitope for Hu antibodies in neurons is the nuclear or cytoplasmatic Hu protein^36^,^37^. Binding of the antibody to an intracellular antigen cannot explain the fast onset excitatory effects because the internalization of antibodies is a slow process^38^. On the other hand, it has been shown that anti-Hu can bind to antigens that are located on the membrane surface of small cell lung cancer cells and neuroblastoma cell lines^39^. However, the function of this membrane bound target or its presence in non-cancer cells is unknown.

The nuclear and cytoplasmatic binding of anti-HuD very likely mediate the long term effects. Our results suggest that the rapid excitatory action of anti-HuD in the ENS involved nicotinic acetylcholine as well as P2X purine receptors; molecular coupling between these two receptors have been described in the ENS^40^,^41^. Both hexamethonium and DHβE blocked nicotinic receptor mediated fast excitatory postsynaptic potentials in the ENS^42^. It is important to emphasize that anti-HuD did not target ionotropic receptors per se. Blockade of the 5-HT_3_ receptor, which is another widely expressed ionotropic receptor in the ENS, did not alter nerve activation by anti-HuD. Our finding that spike discharge evoked by anti-HuD antibody was prevented by the Hu antigen as well as by the HuD fragment aa 90–101 strongly suggested that the excitatory effect depended on the anti-Hu paratope of the antibody, even though the HuD fragment aa 90–101 did not affect the staining. Notably, anti-HuD also activated visceral sensory nerves although we (this study) and others did not observe staining of terminal endings of visceral afferent with any anti-Hu antibody^43^.

The ANNA-1 samples showed strong staining of virtually all neurons in the guinea pig myenteric plexus, while we detected the excitatory effects only in a subpopulation of neurons (median 12% in guinea-pig and 50% in human enteric neurons). Likewise, anti-HuC/D antibodies label all guinea pig and human enteric neurons^9^,^37^ but the anti-HuD activated only a subpopulation of neurons. This suggests that labeling and neuronal activation may occur independently. There was also a mismatch between the number of neurons responding to ANNA-1 (50% in human submucous plexus) and the proportion of neurons functionally expressing the nicotinic receptor (more than 90% based on the occurrence of nicotinic fast EPSPs)^17^. This is unlikely to be related to any lack of sensitivity of voltage sensitive dye recording to detect neuronal activation associated with action potentials discharge since similar responses were obtained using the more sensitive Ca^++^ imaging. The simplest explanation relates to differential affinity or access of ANNA-1 IgGs to ganglionic nicotinic receptors. Alternatively, ANNA-1 IgGs may preferentially activate nicotinic receptors with particular subunit composition. Enteric neurons express α3-, α4-, α3/α5-, β2-, β4- and α7-nicotinic receptor subunits in different heteromeric combinations but all contributed to the excitatory effects of the most important enteric excitatory transmitter acetylcholine^44^. The excitatory effect of ANNA-1 IgGs in the present study was reduced by the β2-subunit preferring antagonist dihydro-β-erythroidine which may suggest some selectivity. However, a contribution of P2X receptor activation is also implicated from the inhibitory action of PPADS. Thus there may be multiple molecular targets for antibody binding and activation. It is unlikely that the nerve-stimulatory effect of the HuD antibody was due to its binding to the intracellular Hu protein for a number of reasons. Firstly, the antibody effect on nerve activation is unrelated to the level of immunostaining. In addition, use of the Basic Local Alignment Search Tool (https://blast.ncbi.nlm.nih.gov/Blast.cgi) to search for homologies with Hu proteins did not reveal any similarity in amino acid sequence to any other known protein, including nicotinic or P2X receptor subunit. However, the antibody binding may only be revealed by modelling the 3D structure of the antibody and the receptor. Secondly, the short latency for neuronal activation suggested an extracellular target. The finding that the response latencies for nicotine and IgGs were similar led us to focus on ionotropic receptor involvement in anti-HuD evoked action potential discharge. This finding combined with the effect of ionotropic receptor antagonists is consistent with nicotinic and P2X receptor involvement. We cannot at this stage fully rule out contribution of metabotropic receptors. However, in our experience spike discharge induced by activation of metabotropic receptors has longer onset latency, at least when using voltage sensitive dye recording. Of course, it is also possible that the primary target of anti-HuD is not a receptor but some structure which is functionally linked to nicotinic and P2X receptors.

It was previously shown that an IgG immune complex formed from mouse IgG as antigen and affinity-purified rat anti-mouse IgG as antibody (but neither antigen nor antibody alone), evoked Ca^++^ signals in dorsal root ganglion neurons through activation of neuronal Fcγ receptor I^45^. However, a possible activation of the Fcγ receptor may be ruled out in our study because the immune complex (through pre-incubation with HuD) had no excitatory effect, but prevented the anti-HuD evoked nerve activation. In addition, there was no Fcγ receptor immunoreactivity in enteric nerve cell bodies (see Fig. 1). We did, however, detect Fcγ receptor immunoreactivity in extrinsic nerve fibers running within the submucous plexus, likely terminals of sensory neurons expressing Fcγ receptor^45^.

Also, anti-HuD antibodies activated mechanosensitive visceral afferents. This finding suggested that a sensitization of mechanosensitive fibers, besides the activation of enteric neurons, may contribute to clinical symptoms related to gut dysfunction in patients with high ANNA-1 titers^46^. The exact link between nerve excitation and symptoms such as pseudoobstruction or gastroparesis remain to be studied. Although our findings do not provide direct evidence it is tempting to speculate that the acute effects of anti-Hu on neuronal excitability (both spike discharge as well as Increase in intracellular Calcium) may with chronic exposure under pathological conditions lead to neuronal damage similar to that observed when primary culture of enteric neurons are exposure for several hours to ANNA-1 sera^8^,^47^.

In summary, we demonstrated excitatory effects of ANNA-1 IgGs on human and guinea pig enteric neurons as well as on mouse visceral afferents. These data suggest that anti-HuD IgG cause direct nerve activation via a mechanism involving nicotinic and P2X receptors. Although the clinical impact of these findings remain to be determined, our data imply that neuronal enteric and afferent excitation triggered by anti-HuD antibodies can play a role in symptom generation in patients with autoimmune-related GI dysfunction.

## Additional Information

**How to cite this article**: Li, Q. *et al*. Anti-Hu antibodies activate enteric and sensory neurons. *Sci. Rep.* **6**, 38216; doi: 10.1038/srep38216 (2016).

**Publisher’s note:** Springer Nature remains neutral with regard to jurisdictional claims in published maps and institutional affiliations.

## Figures and Tables

**Table 1:** Characteristics of patients (ANNA1 samples) and controls.

Patient	Sex	Age	ANNA-1 titer	Total IgG [mg/ml]	Clinical information
#2	M	72	15,360	2.12	Chronic intestinal pseudoobstruction
#3	M	61	61,440	6.27	Smoker, chronic intestinal pseudoobstruction, Small cell lung cancer
#9	F	76	15,360	1.28	Graves disease, chronic intestinal pseudoobstruction
#10	F	67	61,440	1.97	Chronic intestinal pseudoobstruction, small cell lung cancer
#14	M	76	1,22,880	1.8	Chronic intestinal pseudoobstruction, small cell lung cancer
#17	M	76	7,680	5.14	Smoker, chronic obstructive pulmonary disease, Autoimmune gastrointestinal dysmotility, chronic intestinal pseudoobstruction, fatigue syndrome, anorexia
#20	F	44	30,720	11.24	Ex-smoker, subacute sensory neuronopathy. Chronic intestinal pseudoobstruction
#27	F	36	50,000	Not determined	Chronic intestinal pseudoobstruction
